# Social mechanisms of maternal health service use among pregnant women facing intersecting vulnerabilities in Ethiopia: a cross-sectional study

**DOI:** 10.1136/bmjph-2025-002778

**Published:** 2026-05-13

**Authors:** Bee-Ah Kang, Rajiv N. Rimal, Yihunie Lakew, Daryl Stephens, Habtamu Tamene

**Affiliations:** 1 Department of Health, Behavior and Society, Johns Hopkins University Bloomberg School of Public Health, Baltimore, Maryland, USA; 2 Johns Hopkins University Center for Communication Programs, Addis Ababa, Ethiopia

**Keywords:** Public Health, Community Health, Social Medicine, Female, Cross-Sectional Studies

## Abstract

**Introduction:**

Maternal health services, such as antenatal care (ANC) and institutional delivery, may reduce maternal and child health burdens. However, disparities in service coverage persist across Ethiopia. Pregnant women experiencing intersecting vulnerabilities face particularly significant barriers to accessing care. Understanding how social contexts shape maternal care-seeking behaviours can provide valuable insights into supporting underserved pregnant women, offering guidance for future research and practice. This study examines the influence of social and gender norms, as well as spousal relationship dynamics, on service utilisation among this population.

**Methods:**

This study is based on cross-sectional baseline data from a parent quasi-experimental study. Screening tools were developed to identify pregnant women facing vulnerabilities in accessing maternal care. A total of 470 pregnant women participated in the survey, which assessed perceived social norms, gender norms, couple communication, decision-making, social support, ANC visits and institutional delivery experiences. Logistic and linear regression analyses were performed.

**Results:**

Our results showed that descriptive norms, injunctive norms, couple communication and joint decision-making were significantly associated with ANC visits. Descriptive and injunctive norms predicted institutional delivery. ANC visit partially mediated the relationship between social norms and institutional delivery and fully mediated the influence of couple communication on institutional delivery. Our findings also revealed interaction effects among social norms, couple communication and gender norms on health service use.

**Conclusions:**

This study revealed that health service use among underserved pregnant women is heavily influenced by social norms—the behaviours, attitudes and beliefs of people in their social networks. Interpersonal communication with husbands also played a significant role in shaping maternal service utilisation. The findings suggest that future programmes should engage and educate male partners, family members and peer pregnant women to foster a supportive social environment that encourages care-seeking behaviours among new mothers facing intersecting barriers.

**WHAT IS ALREADY KNOWN ON THIS TOPIC**

Attending antenatal care visits and delivering a baby in health facilities can reduce maternal and child health burdens in low- and middle-income countries. While the coverage of these services has increased in Ethiopia, substantial disparities exist. Evidence suggests that social factors, including social norms, social support and interpersonal communication, may promote maternal care-seeking behaviours.

**WHAT THIS STUDY ADDS**

This study illuminated the social factors that predict maternal health service utilisation among pregnant women facing heightened vulnerabilities. Their behaviours were found to be guided by the behaviours and beliefs of other pregnant women and close individuals within their social networks. Pregnant women who frequently engage in respectful communication with male partners are more likely to seek needed care.

**HOW THIS STUDY MIGHT AFFECT RESEARCH, PRACTICE OR POLICY**

This research suggests testing interventions integrating social norms-based messaging, peer modelling or community dialogues and measuring their impact on changing behaviours related to maternal health. Couple-centric approaches may help underserved women access care. Future researchers may consider developing targeted educational interventions for couples, focusing on mutual decision-making, shared responsibilities and fostering supportive communication.

## Introduction

 Ethiopia has achieved substantial strides in maternal and child health over the past decades.^1 2^ The country ranks as one of the top five performers in sub-Saharan Africa, with the maternal mortality ratio decreasing from 871 per 100 000 live births in 2000 to 676 in 2010 and further to 141 in 2025.^3^,^6^ Its success has been attributed to various national policies and programmes that focused on achieving rapid improvements in maternal and child healthcare services.^7 8^ Nevertheless, Ethiopia is still experiencing persistent health challenges. The most recent data indicate that approximately 10 000 mothers die annually, accounting for 3.6% of global maternal mortalities and remaining far above the global target.^9^

This prompts the question of whether efforts to reform the ‘supply-side’ of healthcare are adequately matched by attention to individuals’ experiences and the contextual challenges that impede service utilisation. While institutional delivery (childbirth in health facilities with skilled delivery assistants) may reduce the risk of obstetric complications^10^,^13^ and maternal deaths by 33%,^13 14^ its coverage is not uniform. Evidence shows that 94.8% of women in Addis Ababa delivered in health facilities, while only 23.3% women in Somali did.^2^ Also, women in lower wealth quintiles, with higher parity, and less education were less likely to deliver in health facilities than their counterparts.^2 15^ This indicates that some pregnant women in the country may be experiencing greater disadvantages in seeking maternal care.

It is worth noting that individual behaviours are largely bound by social contexts. This influence is especially pronounced among rural, underserved women whose decision-making is guided by strong social, cultural and familial ties. Indeed, social and cultural beliefs, values, social support and norms have been reported to affect maternal service use in rural communities.^16^,^18^

Social norms, defined as shared rules and expectations that guide and constrain human behaviours within a social network,^19^ can affect maternal behaviours. It was reported that Ethiopian women’s health decisions and birth preparedness plans were strongly influenced by elderly women and opinion leaders in the village.^20 21^ Norms around delivery likely evolved from traditional practices in the community, where the home has long been viewed as a natural, safe and private birthing environment in Ethiopia.^16^ In the social sciences, social norms have been conceptualised as perceptions about others’ beliefs, which can then be discriminated into descriptive norms (beliefs about what is typical and normal based on what others are doing)^19 22^ and injunctive norms (beliefs about what constitutes socially approved conduct).^22^ The theory of normative social behaviour^23^ validated these two distinct types of norms, while suggesting that other contextual factors modulate normative influence.^24^

Meanwhile, the social factors influencing maternal care-seeking behaviours reflect gendered dynamics, as women may encounter gendered expectations during pregnancy. Studies have acknowledged that restrictive gender roles pervasive in society and male decision-making power within households can prevent women from delivering in health facilities.^16 25 26^ Gender norms are a subset of social norms, deeply rooted in institutions, that perpetuate unequal access to resources and power between women and men.^27^ Therefore, it is essential to conceptualise gender norms with an understanding of the institutionalised and systemic realities that shape people’s lives. Descriptive gender norms pertain to beliefs about how frequently other people’s behaviours correspond to the existing gendered system, and injunctive gender norms refer to perceptions about how other people expect one to behave toward the gendered system.^28^

The gendered dynamics extend beyond individual perceptions, manifesting as the actual behaviours within the domestic sphere. Two major forms of gendered contexts that pregnant women may experience in everyday lives can be (1) spousal dynamics and (2) social support. Nussbaum suggested three types of human capability, one of which is combined capabilities—how internal capabilities, together with the external provisions, enable the person to attain desirable bodily health, thoughts and emotions.^29^ Certain social norms and traditions may restrict and violate individuals’ capabilities.^30^ Therefore, women’s access to care can be improved when women are empowered to pursue their goals (ie, making health decisions through communicating with spouse) and simultaneously lift barriers (ie, receiving social support to reduce workloads) that hinder the practical realisation of these capabilities.

Equitable spousal dynamics can be achieved through women’s ability to discuss health and domestic issues with partners and make informed decisions. The frequency and quality of couple communication contributes to women’s access to care. Couple communication was found to improve maternal health behaviours.^31 32^ In Mozambique, women who discussed health seeking with their partners were 46% more likely to deliver in a health facility than those who did not.^33^ Moreover, joint or independent decision-making is likely to ensure access to overall maternal care and reproductive rights and improve health outcomes among women.^34^,^36^ Less than 20% of women participated in health decision-making in sub-Saharan Africa.^37^ While women’s sole decision-making may signify their autonomy and empowerment, it may also imply partners’ lack of engagement in care. Whether women’s empowerment and male involvement are mutually exclusive or reinforcing one another has not been extensively explored; a decision-making process is indeed highly context dependent.

Additionally, pregnant women in rural Ethiopia face the dual burden of heavy workloads from farming and household chores.^38^ Domestic chores are widely seen as women’s sole responsibility during pregnancy, leading many to prefer home births to avoid neglecting essential tasks like childcare.^20 39^ Social support, defined as the perceived or actual resources provided by others, is another important consideration in health research. It has been conceptualised as a multidimensional construct, encompassing informational, instrumental, appraisal and emotional support,^40^ which may partly explain mixed findings across studies. For example, a qualitative study in Ethiopia found that women who had strong social support were susceptible to social persuasion to deliver at home.^41^ Another study in Ghana found that unmarried women who received social support were likely to deliver in health facilities.^42^ Instrumental support may contribute to an understanding of how gendered dynamics in a domestic setting affect women’s access to care. For rural Ethiopian women burdened with extensive workloads,^43^,^45^ such support can facilitate maternal care-seeking behaviours.

While prior studies have examined the social determinants of maternal health service coverage, few have focused on pregnant women experiencing intersecting vulnerabilities. Examining the unique social contexts of this population—such as perceptions of social values, beliefs and gendered dynamics within the household—and the ways in which these factors interact can help shape programme strategies and policies that address their specific needs. Moreover, mounting evidence^1 2 46 47^ suggests that antenatal care (ANC) visit serves as a gateway to institutional delivery; its role in the social pathways leading to institutional delivery remains understudied.

This study addresses research gaps by (1) exploring the relationship between social factors and maternal health service utilisation, (2) examining how ANC visit mediates the relationship between social factors and institutional delivery and (3) analysing the interaction effects of social factors to provide a nuanced understanding of the social mechanisms shaping access to maternal care among underserved pregnant women in Ethiopia.

## Methods

### Study design

Data for this study come from the baseline portion of the parent study that included a first phase (pre-intervention) and a post-intervention second phase. Because data reported in this paper were collected prior to intervention implementation, no participants had been exposed to the intervention at the time of data collection; therefore, baseline data were pooled across intervention and control arms.

The Oromia region was purposefully selected for better demographic and cultural representation. The region is the largest in population size in Ethiopia and geographically shares borders with most regional states, including Amhara, Afar, Benishangul-Gumuz, Gambella, Harari, Sidama, South Ethiopia, South West Ethiopia, Central Ethiopia and Somali regions.

### Sample and recruitment

Screening and recruitment occurred between November and December 2023. Pregnant women between 15 and 49 years old with five or more months of pregnancy and experiencing vulnerabilities were recruited. A pregnancy screening tool and a vulnerability screening tool were developed to identify eligible participants. Women who reported being more than 5 months pregnant proceeded to the vulnerability screening process. The vulnerability screening tool was developed based on a literature review and regression analyses of the 2019 Ethiopian Demographic Health Survey to identify potential factors of maternal health service utilisation among pregnant women. The identified factors were analysed using multivariate cluster analysis (K-medoids) to determine whether they could segment pregnant women into low, moderate and high vulnerability groups. The K-medoids method enabled measuring distance in multiple dimensions, generating meaningful categories or variables. The final tool included economic, social and information and service access dimensions and was pretested to confirm its feasibility. A detailed process of developing the tool is described elsewhere.^48^

The current institutional delivery coverage (48%) in Ethiopia was used as the basis of sample size calculation.^2^ The following assumptions were considered: a p value of 0.05, interclass correlation coefficient of 0.009 to adjust design effect, a power of 80% beta value and a 9% statistical difference expecting at the end of intervention period. This led to a sample size of 480 pregnant women.

With the Central Statistical Agency’s support, this study was permitted to recruit participants from 160 enumeration areas across four study woredas. Within these areas, trained data collectors listed all households with pregnant women, identifying 1614 households for pregnancy screening. Among them, 1189 women were at least 5 months pregnant and completed a vulnerability screening. Of these, 559 women classified as moderately or highly vulnerable constituted the sampling frame. We aimed to recruit approximately three women per enumeration area to achieve target sample size of 480 women. If an enumeration area had five or fewer eligible women, all of them were contacted for recruitment. If there were more than five eligible women in an area, random selection was applied. Recruitment continued until the target sample size was approached; however, complete data were available for 470 participants and included in the analyses. This represents 98% of the planned sample size and is unlikely to affect study validity. Online supplemental appendix 1 describes the overall sampling process.

### Data collection and measures

Data collection was conducted in January 2024. The survey questionnaire included the following key areas: sociodemographics, psychosocial variables (eg, knowledge, attitudes, beliefs, self-efficacy, behavioural intention), social norms, gender norms, spousal dynamics (couple communication and decision-making), social support during pregnancy and maternal service uptake (institutional delivery and ANC visit).

#### Maternal health service utilisation (study outcome)

The primary outcome measure was institutional delivery, which was measured through a question about the place of childbirth for most recent delivery. Participants had options to choose from home, public hospital, health centre, private hospital, health post andnon-governmental organisation (NGO) clinic. The outcome was dichotomised into institutional delivery (coded 1) and non-institutional delivery (coded 0). Health posts were classified as non-institutional delivery facilities, as most lack adequate delivery supplies and skilled birth attendants, following the definition of the WHO^49^ and the guidelines by the Ministry of Health in Ethiopia.^50^ The secondary outcome was ANC visit, based on the self-reported number of ANC visits during the current pregnancy. Those who did not make any visit were coded as 0. The variable was transformed to adjust skewness for analysis.

#### Social norms

Social norms measures were designed to capture both perceived frequency of behaviours and perceived social pressure to conform to the behaviours in the context of general maternal health behaviours. Descriptive norms were assessed based on respondents’ perceived frequency of maternal behaviours in their community. Six items were used to inquire about the expected number of pregnant women engaging in ANC visits, nutritional supplementation, birth preparation and institutional delivery. Responses were on an 11-point scale (0–10 pregnant women) and were averaged to create an index variable. The Cronbach’s α was 0.73.

Injunctive norms were assessed by examining women’s beliefs about how important others expect them to behave regarding specific maternal behaviours. To measure these norms, respondents were first asked to identify the person closest to them from a list of options, including spouse, mother, sister, brother, mother-in-law or other. This approach enabled respondents to select the reference group they felt was most influential to their maternal behaviours. Responses to the six injunctive norms questions were based on a 5-point scale, called the ‘Hand Scale’, a locally adapted Likert scale. The data collector explained the scale using Ethiopian staple food as an example (ie, If you want to prepare injera dough with three portions of teff and 1 portion of sorghum, you will say, ‘I want 3 hands teff and 1 hand sorghum.’) and asked women to indicate their level of agreement with the questions. The Cronbach’s α was 0.83.

#### Gender norms

This study conceptualised gender norms as beliefs about how other people in one’s social network behave and expect one to conform to the unequal division of power between men and women. Given the study’s focus and target population attributes (ie, pregnant women having a husband/male partner), the discussion of gender norms was limited to biologically heterosexual relationships.

The G-NORM scale, which includes descriptive gender norms and injunctive gender norms, has been validated in different contexts.^28^ The reduced G-NORM scale has nine items for each of the two subscales. We adapted this scale to reflect Ethiopian contexts. For example, the item ‘In most families I know, only men are the ones who earn money for the family’ was modified to ‘In most families I know, women are more valued if they do both household and farm work’, taking account of the social convention around women’s dual responsibilities in rural Ethiopia. In addition, four items from the Gender Equitable Men scale were added to incorporate other relevant dimensions of social norms, including reproductive rights (eg, ‘A woman is considered as a real woman only when she has a child’).

All items in the injunctive gender norms scale correspond to those of the descriptive norms scale per recommendations of social norms scholarship.^23 24^ The reference group for this scale was the closest individual to the woman, rather than others in her community, to better assess the social pressure women perceive from those around them. Responses ranged from 0 to 4 to indicate the level of agreement using the Hand Scale. All responses were coded such that a higher score indicated equitable gender norms. The Cronbach’s αs for descriptive and injunctive gender norms were 0.81 and 0.85, respectively.

#### Couple communication

The frequency of communication with spouse/partner was measured with six items for the following topics: family planning, ANC, delivery, iron and folic acid supplementation, nutrition and child health issues using the 5-point Hand Scale. Also, to assess whether interpersonal communication fostered women’s ability to express themselves and ensured mutual respect, the following two questions were used to evaluate their relational dynamics in communication: ‘When you are having a conversation with your partner, do you typically share your opinion?’ and ‘How much do you think your opinion is valued?’ Responses were averaged to create a composite variable (α=0.89).

#### Decision-making

Decision-making was assessed through nine items that asked women about who in the household typically makes decisions on a series of domestic matters and healthcare-seeking behaviours (eg, attending ANC, number of children to have) using the 4-point scale (ie, myself, my husband, both and others). We assigned higher values to joint decision-making, followed by women-only and husband or others-only categories, reflecting evidence that joint decision-making between spouses often leads to better health outcomes than decisions made solely by women without spousal input or agreement.^34 51 52^ This aligns with the rationale that equitable spousal dynamics, fostered through mutual decision-making and communication, enhance women’s empowerment during pregnancy. The Cronbach’s α was 0.87.

#### Social support

Social support was defined as any assistance with household work during pregnancy based on prior evidence that women’s heavy workload was a barrier to accessing health services.^38 43^ In order to measure instrumental support, women were asked to indicate how much their partners or other family helped with their workload during the current pregnancy using two items. Responses were recorded in the 5-point Hand Scale and averaged to create an index variable (*r*=0.61).

#### Covariates

All analyses controlled for age, education, years of residence, family size, engagement of labour, wealth, birth experience and gestational age. These covariates were selected based on their correlation with independent and dependent variables.

### Data analysis

Descriptive analysis was conducted to understand frequencies and percentages of key variables among participants. Continuous variables were examined for normality using histograms. Pearson’s correlation coefficients were estimated among all variables of interest when assumptions of approximate normality and linearity were satisfied. Multivariable logistic regression analyses were conducted for institutional delivery, while linear regression analysis was performed for ANC visit as the outcome variable. Social factors that showed significant associations with the outcome variables in the regression analyses were subsequently included in generalised structural equation modelling, with ANC visit serving as a mediator of each model. Lastly, interaction terms among all social factors were tested using logistic and linear regression analyses to identify social factors’ joint effects on dependent variables. The social factors were mean-centred and standardised prior to interaction modelling, and interaction effects were probed at ±1SD to aid interpretation. This approach improves model stability and reduces multicollinearity between main effects and interaction terms.

## Results

### Participant characteristics

A total of 470 women participated in the survey (table 1). On average, participants were 26.61 years old. Approximately 66% of the women had not received any formal education. The majority of participants were Muslim (91.70%). Most women were living with their spouses (98.48%) and had been residing in the same village for more than 5 years (80.21%). 17% of participants had engaged in income-generating labour in the past year. Out of a maximum possible score of 18 on the wealth index, the mean wealth score was 4.54. Most women (87.66%) had prior childbirth experience and were, on average, 6.61 months pregnant at the time of the survey. The average number of ANC visits made during current pregnancy was 1.46. Among those who had given birth, 47.46% delivered in health facilities.

**Table 1:** Characteristics of pregnant women with intersecting vulnerabilities (n=470)

	M (SD)	%
Demographics		
Age (years)	26.61 (5.65)	
Education		
No education		66.38
Vocational/religious school		0.64
Formal education		32.98
Religion		
Orthodox		5.74
Muslim		91.70
Protestant		2.34
Catholic		0.21
Marital status		
Not married		1.91
Married		98.09
Year of residence		
Less than 1 year		2.55
1–5 years		17.23
More than 5 years		80.21
Family size (number of persons)	5.43 (2.15)	
Cohabitation with spouse*		98.48
Engagement in labour*		17.45
Wealth (number of household assets)	4.54 (2.85)	
Birth experience*		87.66
Gestational age (months)	6.61 (1.21)	
Social factors and health service utilisation		
Descriptive social norms	4.72 (1.83)	
Injunctive social norms	3.20 (1.05)	
Descriptive gender norms	2.71 (0.71)	
Injunctive gender norms	3.25 (0.82)	
Couple communication	22.23 (8.35)	
Joint decision-making	0.98 (0.46)	
Social support	2.35 (1.11)	
ANC visits	1.46 (1.53)	
Institutional delivery*		47.46

* Response indicating yes to respective binary item.

ANC, antenatal care.

Table 2 demonstrates a Pearson’s correlation matrix of key variables involved. The higher the level of education, the greater the reported social norms (γ=0.13, p<0.01 for descriptive norms; γ=0.22, p<0.001 for injunctive norms), equitable gender norms (γ=0.19, p<0.001 for descriptive norms; γ=0.18, p<0.001 for injunctive norms), couple communication (γ=0.23, p<0.001) and social support (γ=0.15, p<0.05). Similarly, wealth was positively related to these social factors. Family size was negatively associated with equitable gender norms (γ=−0.13, p<0.01 for descriptive norms; γ=−0.12, p<0.05 for injunctive norms) and couple communication (γ=−0.11, p<0.05).

**Table 2:** Zero-order Pearson’s correlations among key variables (n=470)

	1.	2.	3.	4.	5.	6.	7.	8.	9.	10.	11.	12.	13.	14.	15.
1. Age	1.00														
2. Education	−0.25^***^	1.00													
3. Years of residence	0.33^***^	−0.11^*^	1.00												
4. Family size	0.53^***^	−0.21^***^	0.23^***^	1.00											
5. Engagement in labour	0.03	0.17^***^	0.02	−0.08	1.00										
6. Wealth	0.10^*^	0.18^***^	0.13^**^	−0.03	0.06	1.00									
7. Birth experience	0.46^***^	−0.19^***^	0.44^***^	0.33^***^	−0.02	−0.01	1.00								
8. Gestational age	−0.03	−0.01	0.00	−0.11^*^	0.01	0.15^**^	−0.05	1.00							
9. Descriptive norms	0.06	0.13^**^	−0.01	−0.04	0.11^*^	0.19^***^	−0.05	0.11^*^	1.00						
10. Injunctive norms	−0.05	0.22^***^	−0.04	−0.09	0.15^*^	0.15^**^	−0.02	0.06	0.46^***^	1.00					
11. Descriptive gender norms	−0.04	0.19^***^	−0.06	−0.13^**^	0.03	0.16^***^	−0.04	0.12^**^	0.27^***^	0.16^***^	1.00				
12. Injunctive gender norms	0.03	0.18^***^	−0.10^*^	−0.12^*^	0.07	0.18^***^	−0.03	0.10^*^	0.31^***^	0.28^***^	0.74^***^	1.00			
13. Couple communication	−0.02	0.23^***^	0.02	−0.11^*^	0.14^**^	0.27^***^	−0.00	0.10^*^	0.37^***^	0.57^***^	0.25^***^	0.26^***^	1.00		
14. Joint decision-making	0.06	0.07	0.07	−0.04	0.01	0.08	0.04	0.02	0.17^***^	0.15^**^	0.17^***^	0.14^***^	0.33^***^	1.00	
15. Social support	0.03	0.15^*^	−0.06	−0.08	0.09^*^	0.22^***^	−0.07	0.15^**^	0.31^***^	0.34^***^	0.15^***^	0.22^**^	0.49^***^	0.12^*^	1.00

*p<0.05; **p<0.01; ***p<0.001.

### Social factors of maternal health service use

Table 3 shows results from regression models with study outcomes. Family size (β=−0.03, SE=0.013, p<0.01) and birth experience (β=−0.19, SE=0.084, p<0.05) were negatively associated, while wealth (β=0.02, SE=0.009, p<0.05) was positively associated with ANC visits. Both descriptive (β=0.07, SE=0.015, p<0.001) and injunctive norms (β=0.07, SE=0.028, p<0.05) were significantly associated with ANC visits along with couple communication (β=0.01, SE=0.003, p<0.01) and joint decision-making (β=0.12, SE=0.044, p<0.01). Family size was negatively associated with institutional delivery (adjusted OR (aOR)=0.88, 95% CI 0.78 to 0.99). Those who perceived higher descriptive norms and injunctive norms were 1.25 times and 1.34 times more likely to deliver in health facilities, respectively (aOR=1.25, 95% CI 1.09 to 1.44; aOR=1.34, 95% CI 1.03 to 1.75).

**Table 3:** Regression models of maternal health service utilisation (n=470)

	Model 1 (ANC visits)	Model 2 (institutional delivery)
	β (SE)	aOR (95% CI)
Demographics		
Age	0.01 (0.004)	0.98 (0.94 to 1.03)
Education	−0.03 (0.027)	1.19 (0.93 to 1.52)
Years of residence	0.10 (0.054)	0.62 (0.34 to 1.13)
Family size	−0.03** (0.013)	0.88* (0.78 to 0.99)
Labour	0.05 (0.061)	0.85 (0.48 to 1.51)
Wealth	0.02* (0.009)	0.99 (0.92 to 1.08)
Birth experience	−0.19* (0.025)	–
Gestational age	0.18*** (0.019)	1.10 (0.92 to 1.31)
Social factors		
Descriptive norms	0.07*** (0.015)	1.25** (1.09 to 1.44)
Injunctive norms	0.07* (0.028)	1.34* (1.03 to 1.75)
Descriptive gender norms	−0.10* (0.049)	0.76 (0.49 to 1.19)
Injunctive gender norms	0.04 (0.043)	1.04 (0.70 to 1.53)
Couple communication	0.01** (0.004)	1.01 (0.97 to 1.05)
Joint decision-making	0.12* (0.044)	0.97 (0.64 to 1.45)
Social support	0.01 (0.024)	1.01 (0.81 to 1.27)

*p<0.05; **p<0.01; ***p<0.001.

ANC, antenatal care; aOR, adjusted OR.

### Mediating role of ANC visits

Regression analyses identified descriptive norms, injunctive norms, couple communication and joint decision-making as predictors of service utilisation. Building on these results, the relationship between these factors and institutional delivery was further examined, with ANC visits included as a mediating variable. The four criteria outlined by Baron and Kenny^53^ were followed for all mediation models. First, the relationship between independent variables (social factors) and dependent variable (institutional delivery) should be significant. Second, the independent variables are significantly associated with the mediator (ANC visits). Third, the mediator is significantly associated with the dependent variable. These results are described in direct paths among the tested variables in table 4. Lastly, the effect of independent variables on dependent variable is significantly reduced (partial mediation) or eliminated (full mediation) when the mediator is included. To test this, the Z-critical value was calculated using Z-transformed correlations and their associated standard errors. The Z-critical score was then compared with standard normal distribution critical values (Z=1.96 for a 95% confidence level) to determine whether the difference in log-odds coefficients between models with and without the mediator was statistically significant. Online supplemental appendix 2 depicts the partial and full mediation models.

**Table 4:** Relationship between social factors and institutional delivery mediated by ANC visit

	Coeff^a^	SE	Z	95% CI	Z_critical^b^_	AIC, BIC
Predictor: descriptive norms						
Direct paths						
Descriptive norms→ANC visit	0.454***	0.077	5.89	0.303 to 0.605		
ANC visit→institutional delivery	1.128***	0.203	5.55	0.730 to 1.527		
Descriptive norms→institutional delivery	0.287***	0.064	4.46	0.161 to 0.413		
Indirect paths						
Descriptive norms→institutional delivery with ANC visit as mediator	0.199**	0.067	2.96	0.531 to 1.364	16.101***	1044.041, 1131.248
Predictor: injunctive norms						
Direct paths						
Injunctive norms→ANC visit	0.603***	0.112	5.40	0.384 to 0.822		
ANC visit→institutional delivery	1.128***	0.203	5.55	0.730 to 1.527		
Injunctive norms→institutional delivery	0.473***	0.107	4.43	0.264 to 0.683		
Indirect paths						
Injunctive norms→institutional delivery with ANC visit as mediator	0.330**	0.114	2.88	0.106 to 0.554	9.90***	1055.487, 1142.695
Predictor: couple communication						
Direct paths						
Couple communication→ANC visit	0.088***	0.016	5.67	0.058 to 0.119		
ANC visit→institutional delivery	1.128**	0.203	5.55	0.730 to 1.527		
Couple communication→institutional delivery	0.039**	0.013	2.91	0.013 to 0.065		
Indirect paths						
Couple communication→institutional delivery with ANC visit as mediator	0.017	0.014	1.18	−0.0111 to 0.04	88.372***	1057.982, 1145.189
Predictor: joint decision-making						
Direct paths						
Joint decision-making→ANC visit	0.817***	0.201	4.05	0.422 to 1.211		
ANC visit→institutional delivery	1.128**	0.203	5.55	0.730 to 1.527		
Joint decision-making→institutional delivery	0.123	0.186	0.66	−0.242 to 0.488		

a Log-odds coefficients.

bZ_critical_= Z1−Z2 √𝑆𝐸1 2+𝑆𝐸2 2, where Z1 is the z-transformed value of the correlation between the independent variable and the dependent variable without the mediator, and Z2 is the z-transformed value of the correlation between the independent variable and the dependent variable with the mediator included in the model.

*p<0.05; **p<0.01; ***p<0.001

AIC, Akaike Information Criterion; ANC, antenatal care; BIC, Bayesian Information Criterion.

The relationship between descriptive norms and institutional delivery was partially mediated by ANC visit. The indirect effect of descriptive norms on institutional delivery, mediated by ANC, remained positive and significant (Z_c_=16.10, p<0.001), indicating partial mediation. The relationship between injunctive norms and institutional delivery also remained significant after including ANC visit as a mediator (Z_c_=9.90, p<0.001). Further, the results show that the relationship between couple communication and institutional delivery was fully mediated by ANC visit. When including the mediators, the indirect effect of couple communication on institutional delivery was eliminated (Z_c_=88.37, p<0.001). Lastly, although joint decision-making was significantly associated with ANC visit (β=0.82, SE=0.201, p<0.001), subsequent mediation tests were not conducted since the independent variable was not significantly associated with the dependent variable.

### Moderation effects of social factors

#### Interaction between descriptive norms and injunctive norms

**Figure 1:**
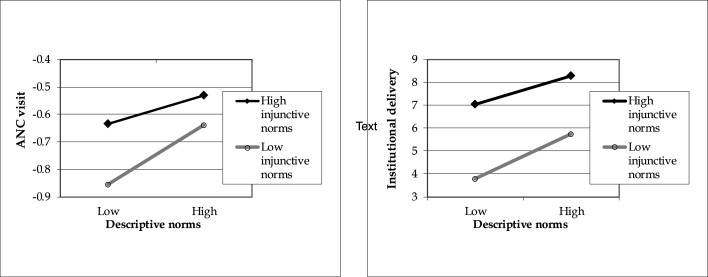
Relationship between descriptive norms and maternal health service use moderated by injunctive norms. ANC, antenatal care.

Interaction effects between descriptive norms and injunctive norms on maternal service utilisation were tested (see online supplemental appendix 3). Both models showed significant interaction effects (β=−0.03, SE=0.011 for ANC visit; aOR=0.89, 95% CI 0.79 to 0.99 for institutional delivery). Figure 1 demonstrates that women who perceived both descriptive and injunctive norms as high exhibited better maternal health behaviours. Although women with low injunctive norms showed lower overall use of ANC and institutional delivery, increases in descriptive norms had a stronger effect for this group, suggesting that descriptive norms strongly amplify care-seeking when injunctive norms are weak.

#### Interaction between social norms and couple communication

Regression analysis results with interaction terms between descriptive norms and couple communication are reported. Only in the model with ANC visit as outcome, the interaction relationship was statistically significant (β=−0.00, SE=0.001) (see online supplemental appendix 4). Women who perceived high descriptive norms and reported frequent, respectful communication with their spouses were the most likely to attend ANC visits. Among women with low levels of couple communication, higher descriptive norms corresponded with notable increases in ANC uptake (see online supplemental appendix 5). The interaction relationship between injunctive norms and couple communication was not significant.

#### Interaction between gender norms and couple communication

Interaction terms between descriptive gender norms and couple communication were explored (see online supplemental appendix 6). This interaction effect was statistically significant only on institutional delivery (aOR=0.96, 95% CI 0.93 to 1.00). Similarly, the interaction effect between injunctive gender norms and communication was statistically significant only on institutional delivery (aOR=0.96, 95% CI 0.93 to 0.99) (see online supplemental appendix 7). Even women who are generally less engaged in couple communication are more likely to deliver in health facilities when they perceive that other families uphold equitable gender norms (descriptive norms) and feel social pressure from close referents to do the same (injunctive norms). The magnitude of these interaction effects, however, appeared weak (see online supplemental appendix 8).

## Discussion

This study conducted a comprehensive test of the role of social factors in maternal healthcare access among pregnant women facing heightened vulnerabilities in rural Ethiopia. We found that both descriptive and injunctive norms were significantly associated with ANC visit and institutional delivery. This suggests that when women perceive other pregnant women in their community using maternal health services, they are more likely to do the same. Additionally, individuals close to pregnant women play a critical role in shaping their behaviours. These results align with previous studies highlighting the influence of social norms on maternal and reproductive behaviours in India,^54^ Bangladesh^55^ and the Democratic Republic of Congo.^56^ Behavioural attributes concerning pregnancy can partly explain these results. Rimal and Lapinski^24^ argued that ambiguity about the appropriate behaviour can amplify normative influence. When the government’s promotion of institutional delivery conflicts with traditional home birth practices, pregnant women may rely on social cues to navigate the uncertainty created by these conflicting messages.

Interaction effects between descriptive and injunctive norms further revealed that women who perceived both norms strongly were most likely to attend ANC and deliver in health facilities. This also suggests that even women who have not previously sought care due to low social expectations from close individuals (injunctive norms) are likely to change their behaviour once they perceive other pregnant women using these services (descriptive norms), effectively aligning with the behaviour of those in the high injunctive norms group. Emphasising both types of norms through targeted counselling messages and intervention strategies is crucial. For instance, organising group-based education for pregnant women and involving close individuals—such as husbands, mothers and siblings—in care throughout the pregnancy journey could enhance the effectiveness of programmes and policies. A recent study found that a social norms-based intervention was effective in improving nutritional behaviors among women of reproductive age.^57^

Our data showed a partial mediating role of ANC in the relationship between social norms and institutional delivery. This partial mediation suggests that social norms are associated with institutional delivery through additional factors beyond the scope of this study. We recommend future research to investigate these factors to better inform strategies for improving institutional delivery among women less likely to attend ANC. In particular, exploring potential mediators of the relationship between descriptive norms and institutional delivery may be especially valuable, given its stronger model fit compared with other social factors.

Meanwhile, promoting couple communication about maternal health issues is a key consideration for future practice. Notably, couple communication was significantly associated with ANC visits among pregnant women. Women who openly communicated and felt respected by their partners during such interactions were more likely to seek timely care. This is in line with a systematic review that found that interventions engaging men in maternal and child health showed more equitable couple communication and decision-making about health and increased care-seeking behaviours.^58^ Male involvement is especially important in patriarchal settings where men control resources and make decisions about health-seeking behaviours for their wives, yet often view pregnancy as solely a women’s issue. However, we acknowledge that shifting gender roles is a challenging endeavour, making incremental strategies potentially more effective. Programmes could offer couple discussion sessions, providing opportunities for partners to collaboratively address solutions for their baby’s health. Additionally, engaging male peers and community opinion leaders can encourage male involvement in maternal care, reducing resistance from male partners and fostering broader acceptance.

Couple communication was significantly associated with institutional delivery, with ANC visits fully mediating this relationship. Our findings suggest that increasing institutional delivery rates simultaneously necessitates strengthening ANC services. Evidence revealed that women’s lack of trust in health systems and experiences of disrespectful care and abusive treatment by health workers prevented them from giving birth in health facilities.^41 59 60^ Mistreatment in maternal care often arises from conflicts between medical practices and cultural traditions preferred by women.^61 62^ It is imperative that ANC providers deliver respectful counselling for pregnant women, dedicating adequate time to address women’s concerns and uncertainties about delivering in health facilities.

Additionally, it is important to note that both descriptive and injunctive gender norms interacted with couple communication in influencing institutional delivery, but not ANC attendance. When women talked openly with their partners and perceived that other families and close referents endorsed equitable gender norms, their likelihood of delivering in a facility increased. These findings underscore fundamental differences between the two maternal health behaviours. ANC visits, linked to a wider range of social factors in our data, may be more amenable to change; they are routine and comparatively less disruptive to gender expectations. Institutional delivery, by contrast, is a single, resource-intensive event that demands joint decision-making, logistical planning and a departure from entrenched gender norms—particularly in contexts where women are often excluded from financial and household decision-making. Thus, interventions that simultaneously foster equitable community gender norms and strengthen couple communication could be pivotal for improving facility-based delivery, whereas ANC uptake may respond to a broader set of social influences. These hypotheses, however, warrant further investigation into whether and how gender norms interact with spousal dynamics in the context of maternal care. Such research could guide future strategies to promote equitable gender norms in ways that align with women’s domestic settings and cultural values.

Another important contribution of this study is the finding that women with larger family sizes, lower wealth indices and prior birth experience were less likely to use maternal health services. Prior studies have also illustrated that these factors served as barriers to maternal care in Ethiopia.^63 64^ Women with high parity may be overburdened with childcare responsibilities, leaving them with less capacity to seek care for themselves. It may also be difficult to leave children in the care of others, hence they may decide to deliver at home. Additionally, extreme poverty may intensify concerns about transportation costs to distant health facilities, further compounded by lost income opportunities during travel and hospitalisation. We recommend involving male partners and family members of this population to establish support systems, such as sharing domestic chores and planning birth preparations, including travel financing. Strengthening domestic support can ultimately shift community norms, fostering an environment that empowers underserved pregnant women to seek proper care.

## Limitations

This study has several limitations. Although many of the social factors tested pertained to spousal dynamics and perceived expectations from close individuals, we did not survey male partners or other family members. A rigorous examination of perceptions, attitudes and knowledge among these individuals will guide future programmes aiming for a holistic shift of the social environment. We acknowledge potential sampling biases, as our study sites and participants were not randomly selected. This decision was influenced in part by the ongoing security challenges in the country, which restricted our ability to conduct random sampling. Nevertheless, considering the inherently selective eligibility criteria of our participants, we believe our approach sheds light on context-specific strategies designed to support underserved pregnant women. Also, despite adjusting for a range of sociodemographic and reproductive characteristics, the possibility of residual confounding remains. Factors that were not captured in our dataset—such as exposure to community health promotion activities, distance to health facilities and prior experiences with the health system—may influence both the social factors examined and maternal health service use. Future studies incorporating broader levels of covariates may help isolate the independent effects of social factors on ANC attendance and institutional delivery. Furthermore, some measures of social determinants may encounter validity challenges. Specifically, the injunctive norms measure focuses on an individual’s perception of a single reference point, despite norms often being shaped by multiple sources. Future research could delve into the heterogeneity of social norms to address this limitation.

## Conclusion

Pregnant women facing intersecting vulnerabilities often encounter significant barriers to accessing and using essential maternal health services. Our study found that their perceptions of other pregnant women’s behaviours and beliefs about the expectations of close references are strongly linked to their care-seeking behaviours. Interpersonal communication with male partners also emerged as a critical factor. This research is among the few studies exploring how social drivers influence health behaviours among underserved Ethiopian pregnant women. It provides valuable insights for designing tailored strategies to address this group, ultimately bridging gaps in maternal health service coverage in Ethiopia.

## Supplementary material

10.1136/bmjph-2025-002778 online supplemental file 1

## Data Availability

Data are available upon reasonable request.
