# Supplementary material for: Social mechanisms of maternal health service use among pregnant women facing intersecting vulnerabilities in Ethiopia: a cross-sectional study
Source: BMJ Public Health. 2026 May 13;4(2):e002778. doi: 10.1136/bmjph-2025-002778 (PMC13182290; doi:10.1136/bmjph-2025-002778)
Supplement: online supplemental file 1 [file bmjph-4-2-s001.docx]

**[Supplementary materials]**

**Appendix 1. Sampling flow diagram**

**Appendix 2. Mediation models**

**
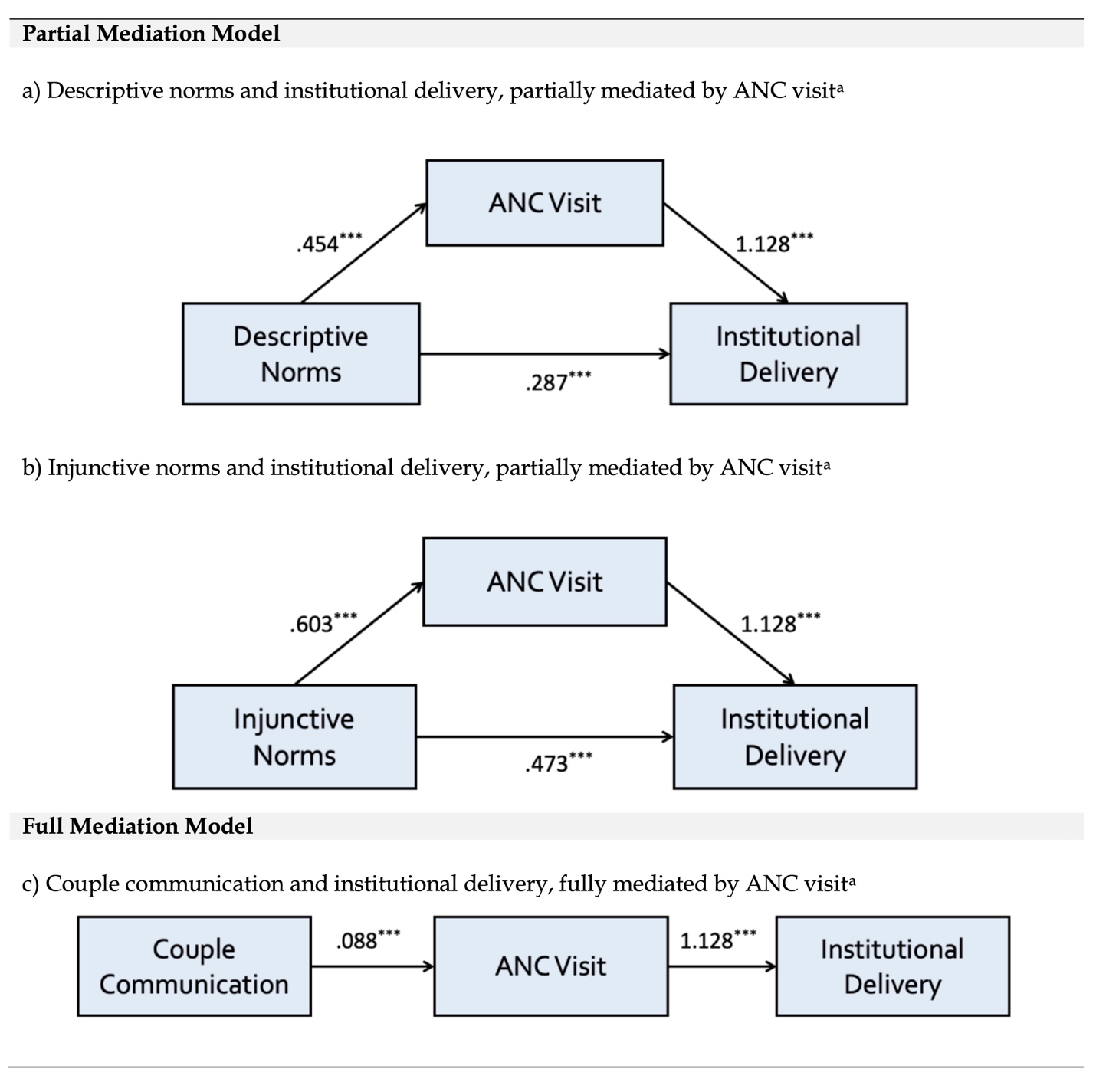
**

**Appendix 3. Regression models with interaction between descriptive norms and injunctive norms**

|  | *Model 1*  *(ANC visit)* | *Model 2*  *(Institutional delivery)* |
| --- | --- | --- |
|  | β (SE) | aOR (95% CI) |
| *Demographics and covariates* |  |  |
| Age | .01 (.01) | .99 (.94, 1.04) |
| Education | -.02 (.03) | 1.19 (.93, 1.52) |
| Years of residence | .11 (.06) | .62 (.34, 1.11) |
| Family size | **-.4^**^** (.01) | **.88^*^** (.78, .999) |
| Labor | .07 (.06) | .88 (.50, 1.55) |
| Wealth | **.03^**^** (.01) | .99 (.91, 1.07) |
| Birth experience | -.19^*^ (.09) | - |
| Gestational age | **.18^***^** (.02) | 1.12 (.94, 1.33) |
| *Main effects* |  |  |
| Descriptive norms | **.18^***^** (.04) | **1.87^**^** (1.22, 2.86) |
| Injunctive norms | **.24^**^** (.05) | **2.36^**^** (1.35, 4.11) |
| *Interaction effects* |  |  |
| Descriptive norms x Injunctive norms | **-.03^*^** (.01) | **.89^*^** (.79, .99) |

**Appendix 4. Regression models with interaction between descriptive norms and couple communication**

|  | *Model 1*  *(ANC visit)* | *Model 2*  *(Institutional delivery)* |
| --- | --- | --- |
|  | β (SE) | OR (95% CI) |
| *Demographics and covariates* |  |  |
| Age | .01 (.01) | .98 (.94, 1.03) |
| Education | -.03 (.03) | 1.19 (.93, 1.51) |
| Years of residence | .09 (.05) | .62 (.35, 1.12) |
| Family size | **-.03^**^** (.01) | .89 (.79, 1.01) |
| Labor | .07 (.06) | .91 (.52, 1.58) |
| Wealth | **.02^*^** (.01) | .99 (.91, 1.07) |
| Birth experience | **-.19^*^** (.08) | - |
| Gestational age | **.18^***^** (.02) | 1.08 (.91, 1.29) |
| *Main effects* |  |  |
| Descriptive norms | **.20^***^** (.04) | **1.48^*^** (1.04, 2.12) |
| Couple communication | **.04^***^** (.01) | 1.05 (.98, 1.13) |
| *Interaction effects* |  |  |
| Descriptive norms x Couple communication | **-.00^**^ (**.00) | .99 (.98, 1.01) |

**Appendix 5. Relationship between descriptive social norms and ANC visit moderated by couple communication**

**Appendix 6.** **Regression models with interaction terms between descriptive gender norms and couple communication**

|  | *Model 1*  *(ANC visit)* | *Model 2*  *(Institutional delivery)* |
| --- | --- | --- |
|  | β (SE) | OR (95% CI) |
| *Demographics and covariates* |  |  |
| Age | .01 (.01) | .99 (.94, 1.04) |
| Education | -.02 (.03) | 1.21 (.96, 1.53) |
| Years of residence | .09 (.06) | .59 (.33, 1.05) |
| Family size | **-.04^**^** (.013) | .89 (.79, 1.01) |
| Labor | .08 (.06) | .94 (.54, 1.63) |
| Wealth | .03^**^ (.01) | 1.02 (.94, 1.10) |
| Birth experience | -.22^*^ (.09) | - |
| Gestational age | **.18^***^** (.02) | 1.09 (.92, 1.29) |
| *Main effects* |  |  |
| Descriptive gender norms | .02 (.10) | 2.02 (.87, 4.69) |
| Couple communication | **.03^*^** (.01) | **1.15^**^** (1.04, 1.27) |
| *Interaction effects* |  |  |
| Descriptive gender norms x Couple communication | -.00 (.00) | **.96^*^** (.93, 1.00) |

**Appendix 7.** **Regression models with interaction terms between injunctive gender norms and couple communication**

|  | *Model 1*  *(ANC visit)* | *Model 2*  *(Institutional delivery)* |
| --- | --- | --- |
|  | β (SE) | OR (95% CI) |
| *Demographics and covariates* |  |  |
| Age | **.01^**^** (.01) | .99 (.95, 1.04) |
| Education | -.02 (.03) | 1.20 (.94, 1.52) |
| Years of residence | .09 (.06) | .60 (.33, 1.07) |
| Family size | **-.03^*^** (.01) | .88 (.78, 1.00) |
| Labor | .08 (.06) | .93 (.53, 1.61) |
| Wealth | **.02^**^** (.01) | 1.02 (.94, 1.10) |
| Birth experience | **-.22^*^** (.09) | - |
| Gestational age | **.18^***^** (.02) | 1.09 (.92, 1.29) |
| *Main effects* |  |  |
| Injunctive gender norms | .09 (.057) | **2.43^*^** (1.20, 4.95) |
| Couple communication | **.02^**^** (.009) | **1.20^**^** (1.07, 1.33) |
| *Interaction effects* |  |  |
| Injunctive gender norms x Couple communication | -.00 (.002) | **.96^**^** (.93, .99) |

**Appendix 8. Relationship between gender norms and institutional delivery, moderated by couple communication**
